# Application of Solution Method to Prepare High Performance Multicomponent Oxide Thin Films

**DOI:** 10.3390/membranes12070641

**Published:** 2022-06-22

**Authors:** Yaru Pan, Xihui Liang, Zhihao Liang, Rihui Yao, Honglong Ning, Jinyao Zhong, Nanhong Chen, Tian Qiu, Xiaoqin Wei, Junbiao Peng

**Affiliations:** 1 Institute of Polymer Optoelectronic Materials and Devices, State Key Laboratory of Luminescent Materials and Devices, South China University of Technology, Guangzhou 510640, China; 201930173424@mail.scut.edu.cn (Y.P.); 201530291443@mail.scut.edu.cn (Z.L.); yaorihui@scut.edu.cn (R.Y.); 202010103138@mail.scut.edu.cn (J.Z.); chen-nanhong@foxmail.com (N.C.); psjbpeng@scut.edu.cn (J.P.); 2 Institute of Semiconductors, Guangdong Academy of Sciences, Guangzhou 510650, China; liangxh@hotmail.com; 3 Department of Intelligent Manufacturing, Wuyi University, Jiangmen 529020, China; 4 Southwest Institute of Technology and Engineering, Chongqing 400039, China; weixiaoqin810913@163.com

**Keywords:** zirconium-yttrium-magnesium-aluminum-hafnium-oxide, dielectric layer, metal oxide film, solution method

## Abstract

Capacitors play an increasingly important role in hybrid integrated circuits, while the MIM capacitors with high capacitance density and small thickness can meet the needs of high integration. Generally speaking, the films prepared with a single metal oxide dielectric often achieve a breakthrough in one aspect of performance, but dielectric layers are required to be improved to get better performance in leakage current, capacitance density, and transmittance simultaneously in modern electronic devices. Therefore, we optimized the performance of the dielectric layers by using multiple metal oxides. We combined zirconia, yttria, magnesium oxide, alumina, and hafnium oxide with the solution method to find the best combination of these five metal oxides. The physical properties of the multi-component films were measured by atomic force microscopy (AFM), ultraviolet-visible spectrophotometer, and other instruments. The results show that the films prepared by multi-component metal oxides have good transmittance and low roughness. The thicknesses of all films in our experiment are less than 100 nm. Then, metal–insulator–metal (MIM) devices were fabricated. In addition, we characterized the electrical properties of MIM devices. We find that multi-component oxide films can achieve good performances in several aspects. The aluminum-magnesium-yttrium-zirconium-oxide (AMYZO_x_) group of 0.6 M has the lowest leakage current density, which is 5.03 × 10^−8^ A/cm^2^ @ 1.0 MV/cm. The hafnium-magnesium-yttrium-zirconium-oxide (HMYZO_x_) group of 0.8 M has a maximum capacitance density of 208 nF/cm^2^. The films with a small thickness and a high capacitance density are very conducive to high integration. Therefore, we believe that multi-component films have potential in the process of dielectric layers and great application prospects in highly integrated electronic devices.

## 1. Introduction

In recent years, MIM capacitors have been widely applied in various electronics, such as dynamic random-access memory (DRAM), flexible electronics [1], super-capacitor, and micro-electro-mechanical systems (MEMS) resonators [2]. In order to meet the miniaturization and high integration of devices, a higher capacitance density is required [2,3]. Therefore, to improve the capacitance density of the MIM capacitor, the researchers use high-k materials as insulators [4]. High-k dielectrics are of great significance for obtaining low leakage current and high capacitance density simultaneously at a small size of a nanoscale [5].

Metal oxides are considered one of the ideal options for gate dielectric materials because of their high transmittance, good uniformity, and excellent electrical stability [6,7,8]. So, metal oxide films are used to make microelectronic devices [9,10]. The fabrication of metal oxide films initially relies on vacuum-based methods (e.g., sputtering and chemical vapor deposition), which require long-term treatment in high vacuum environments to deposit the films successfully. Vacuum methods also require post-deposition treatment at a relatively high temperature for membrane densification [11]. However, vacuum deposition is difficult to satisfy the request for low-cost and large-scale production. This severely limits their potential in practical applications [12]. With the rapid development of film deposition technology, the recent use of solution-based processes alleviates the shortcomings of the high cost of the vacuum method, which is unsuitable for industrial development. Since the vacuum method for preparing thin films has been very mature, the leakage current of the MIM capacitor can be less than 10^−8^ A/cm^2^ @ 1.0 MV/cm [13,14]. However, the leakage current of film devices fabricated by the solution method can only reach less than 10^−6^–10^−7^ A/cm^2^ @ 1.0 MV/cm [15,16]. Therefore, the preparation of metal oxide films with excellent properties by solution method at low temperature has attracted extensive attention from researchers [12,17,18,19]. Solution-based manufacturing significantly reduces manufacturing costs by eliminating vacuum deposition processes and replacing them with printable precursor materials. Additionally, compared with the traditional method, solution processing is easier to operate and regulate the chemical composition, contributing to the high quality of thin films [20,21].

According to previous research results, we find that the single-metal or binary-metal oxide films working as the dielectric layer did make great progress in leakage current density or permittivity, such as Al_2_O_3_ [22], ZrO_2_ [23], HfO_2_ [24], Y_2_O_3_ [25], and HfLaO [26], but these systems focus on making a breakthrough in one aspect. Therefore, to optimize different properties of the dielectric layers simultaneously, we combined multi-component metal oxides. Previously, our team has studied the performance of amorphous zirconium-yttrium-aluminum-magnesium-oxide (ZYAMO) metal oxide films [27]. In the studies, we found that the films prepared by mixing four metal oxides can balance the relation between relative dielectric constant and bandgap. Therefore, aiming to find if a better performance can be obtained under other conditions, we compared various multi-component metal oxides. This experiment will further explore the possibility of other metal oxide combinations to optimize the performance of dielectric layers in thin-film devices. N. Wu and Q. C. Zhang found that HfO_2_ film has better interface quality, so it has been actively studied as the gate dielectric of transistors [28]. At the same time, hafnium oxide has a high dielectric constant (~30) and large bandgap (~5.68 eV) [29], which enables the devices to obtain better capacitor performances. In addition, studies have shown that hafnium oxide films can achieve lower leakage currents than other oxide films at similar capacitance densities [30,31]. Therefore, based on a quaternary combination, we added hafnium oxide to explore further the potential of the multi-component metal oxides. We combined five kinds of metal to prepare polyoxide precursors with different concentrations and carried out research on the good optical and electrical properties of multi-component oxide films.

As a result, the aluminum-magnesium-yttrium-zirconium-oxide (AMYZO) group of 0.6 M has the lowest leakage current density of 5.03 × 10^−8^ A/cm^2^ @ 1.0 MV/cm. The hafnium-magnesium-yttrium-zirconium-oxide (HMYZO) group of 0.8 M has a maximum capacitance density of 208 nF/cm^2^. The aluminum-hafnium-magnesium-yttrium-oxide (AHMYO) group component with the best optical properties has an average transmittance of 95%. Compared with the oxide films prepared by the solution method, the multiple oxide films in this experiment have made some breakthroughs in leakage current and capacitance density [15,32]. Above all, the films in this experiment have great application prospects for making transparent new electronic devices with good transmittance, low leakage current, and high capacitance density.

## 2. Materials and Methods

Zirconium nitrate (Zr(NO_3_)_4_·5H_2_O), yttrium nitrate hexahydrate (Y(NO_3_)_3_·6H_2_O), magnesium acetate tetrahydrate (C_4_H_6_MgO_4_·4H_2_O), aluminum nitrate nonahydrate (Al(NO_3_)_3_·9H_2_O), and hafnium chloride (HfCl_4_) were dissolved in ethylene glycol monomethyl ether (EGME) to prepare precursors with three concentrations (0.6 M/0.8 M/1.0 M). The molar ratio of five metal salts is 1:1:1:1:1. To suppress the hydrolysis of metal ions, we added acetic acid before stirring the solutions in the atmosphere for 8 h. After that, we filtered them with a 13 mm/0.45 μm filter and then aged the precursor for 24 h.

So as to increase the adhesion of precursors to substrates, the substrates were treated with Plasma for five minutes before spin coating. Then, 50 μL of the precursors was spin-coated on the glass substrates at 1000 r/min for 6 s and 5000 r/min for 30 s to obtain thin films. After pre-annealing at 130 °C, the films were annealed at 300 °C for 1.5 h in the air.

To describe the groups briefly, we named the six combinations as follows (Table 1).

We measured the surface tension and the contact angle with the ITO substrates of precursors by an Attension Theta Lite (TL200, BiolinScientific, Gothenburg, Sweden). We tested the viscosity by rheometer for rheological properties (TL200, BiolinScientific, Gothenburg, Sweden). The surface features of the films were observed with a laser scanning confocal microscope (LSCM, OLS50-CB, Tokyo, Japan). The surface morphologies of the dielectric layer films were observed by atomic force microscopy (AFM, BY3000, Nano Instruments, Guangzhou, China). A thermogravimetric (TG) analyzer was used to analyze the thermal behaviors of the precursors at a heating rate of 10 °C/min from room temperature to 400 °C. The chemical groups in the films were investigated by Fourier transform infrared spectroscopy (FTIR, ATR Accessory, Nexus, Madison, WI, USA). A step profiler (Dektak150, Veeco, Tucson, AZ, America) was used to test the thickness of the multiple oxide films. The optical properties of the films were studied by a UV-Vis spectrophotometer (UV-VIS, UV-3600 Shimadzu, Kyoto, Japan).

The current–voltage (I–V) and capacitance–voltage (C–V) characteristics of MIM devices were measured by the Keithley 4200 (Tektronix, Beaverton, OR, USA) parameter analyzer under atmospheric ambient conditions.

## 3. Results and Discussion

### 3.1. Solution Properties and Optical Properties

**Figure 1:**
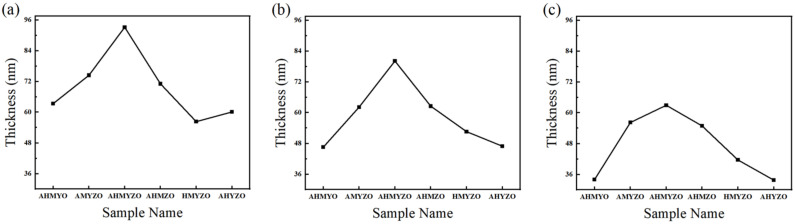
(**a**) The thickness of 1.0 M; (**b**) the thickness of 0.8 M; (**c**) the thickness of 0.6 M.

We measured the thickness of the films after annealing, and the results are shown in Figure 1. There is a positive correlation between thickness and concentration under the same composition. As shown in Figure 1, the thickness of the thin film increases as concentration rises. Material supply influenced by the concentration affects the growth process of the thin films. When the concentration increases, sufficient solute molecules accumulate quicker to grow thicker films [33]. Based on the findings of previous studies, the thickness is mainly affected by two mechanisms during spin coating, including convective radial outflow and solvent evaporation. Two mechanisms work together in the first stage. At the same time, the second stage is mainly affected by the solvent evaporation mechanism which is generally related to the property and molecular weight of the solute [34,35]. We find that at the same concentration, the thickness of different films varies greatly. In addition, from Figure 1, we find that the relationship between thickness and element composition is almost the same at three concentrations. We speculate that the relationship between elements will not change with the increase in concentration. Under the same concentration, the thickness of the AHMYZO group is the largest. This result might be ascribed to the interaction between the metal atoms, leading to the loose internal structure of the films. As can be seen in Figure 1, the thicknesses of the AHYZO group are smaller in comparison. This might be attributed to a bigger radius of the magnesium ions, though its molecular weight is the smallest. Therefore, the compactness of the film will decrease when magnesium ions are added. Therefore, we speculate that the films of the AHYZO group are dense, which affects the electrical properties of thin films. In conclusion, the thicknesses of the films are influenced by the concentration and molecular weight. A higher concentration or larger molecular weight comes down to a thicker film.

**Figure 2:**
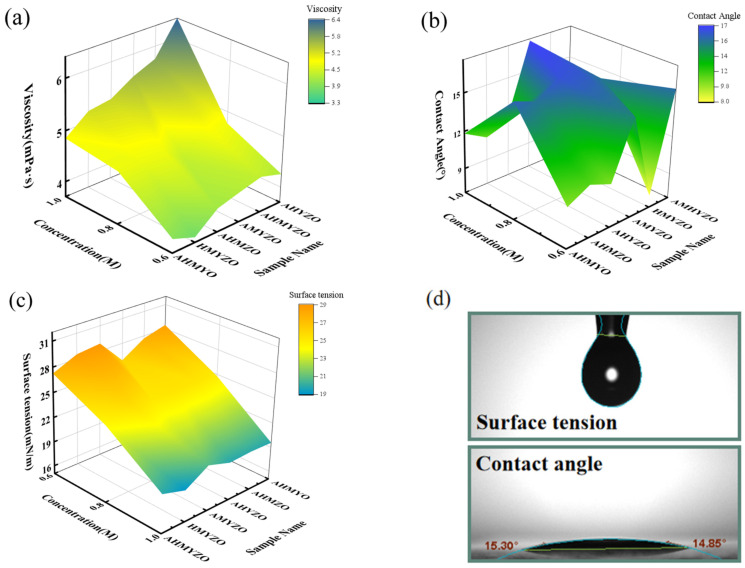
(**a**) The viscosity of the precursor solution of different precursors; (**b**) contact angles of different precursors on ITO substrate; (**c**) surface tension of different precursors; (**d**) schematic diagram of contact angle and surface tension measurement.

Then, we tested the viscosity, the surface tension, and the contact angle of precursors since they are the key factors for wettability. The results are shown in Figure 2a–c. Additionally, Figure 2d shows the schematic diagram of contact angle and surface tension measurement. The viscosity grows up as the concentration rises, which may be due to an enlargement in the internal friction of atoms, increasing viscosity. In Figure 2c, it can be observed that the surface tension decreases as the concentration rises, owing to the decline of the intramolecular force of the solvent molecule [36,37]. Comparing the results in surface tension and viscosity, we find that the contact angles varied a lot. In terms of the contact angles, there is no obvious trend with the change in the concentrations. We speculate that it is probably ascribed to the properties of the solute. More formation of hydrogen bonds increases the surface energy of the liquid and enhances the wettability [38]. For example, the C-O-Hf hydrogen bond is formed among hafnium ions and acetic acid. These bonds lead to the rise of wettability, resulting in larger contact angles of the AMYZO groups [39]. Therefore, the contact angles of the precursors with hafnium oxide are smaller than that of AMYZO groups. Generally speaking, suitable physical properties of the solutions are the basis of gaining uniform thin films by spin coating. According to the test results, the viscosity, the surface tension, and the contact angle are of suitable values, indicating the solutions have great wettability on the surface of the ITO substrate [40,41].

**Figure 3:**
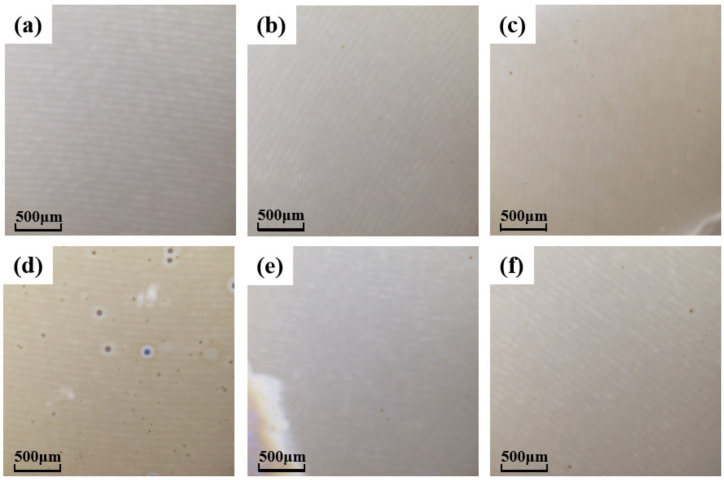
Laser scanning confocal microscopy images of thin films with a concentration of 1.0 M (**a**) AHMYO film; (**b**) AHMZO film; (**c**) AHYZO film; (**d**) HMYZO film; (**e**) AMYZO film; (**f**) AHMYZO film.

In order to characterize the surface morphology of the thin films, we observed the morphological characteristics of the films under different concentrations with a laser scanning confocal microscope. The results are shown in Figure 3. Except for the HMYZO film at 1.0 M, the surfaces of other films are flat without obvious particles. This is probably the result of aluminum oxide playing a role in regulating the arrangement of metal atoms during the growth of the films [18]. That is why the films of the HMYZO group precipitate at high concentrations.

**Figure 4:**
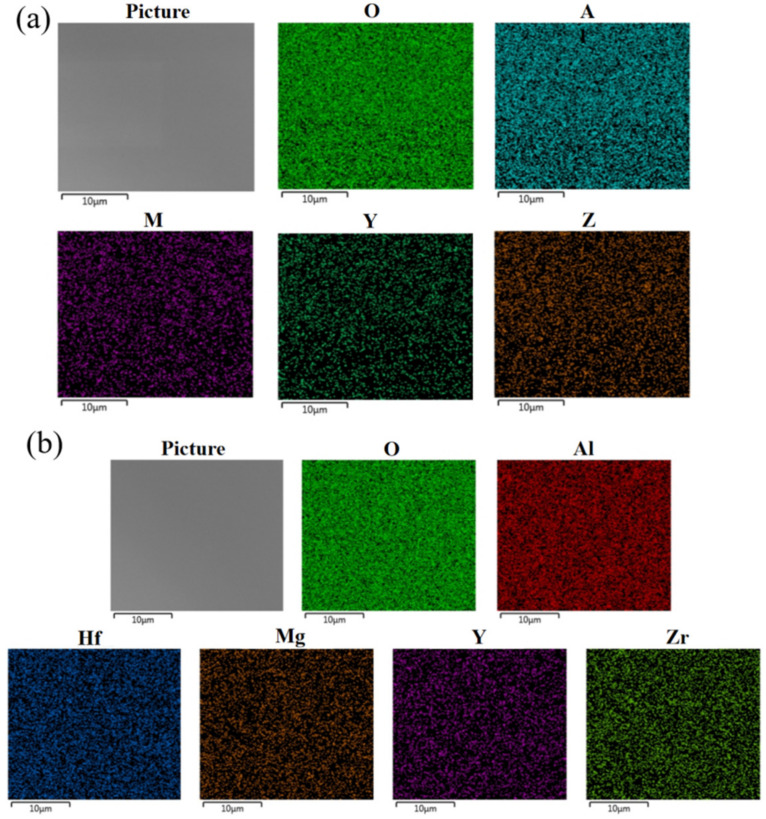
(**a**) EDS diagram of AMYZO group; (**b**) EDS diagram of AHMYZO group.

In order to reflect the distribution of elements in the films, we gained the EDS pictures of the films by SEM (scanning electron microscopy). The EDS image results are shown in Figure 4. The results of EDS showed that the elements were uniformly distributed. Moreover, we can see that the distribution density of each element in the same region is roughly the same, which can be approximately regarded as the uniform distribution of metal elements.

**Figure 5:**
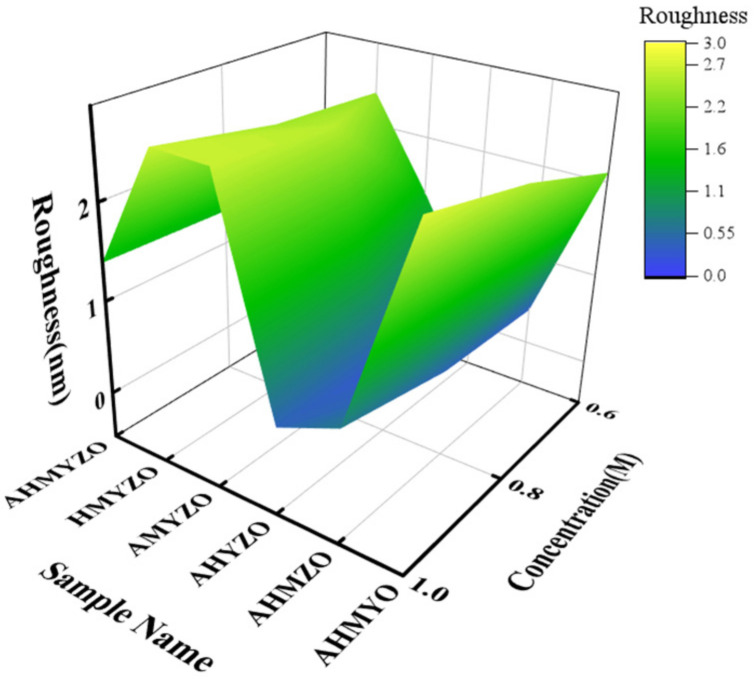
Surface roughness of films from 0.6 M to 1.0 M group.

**Figure 6:**
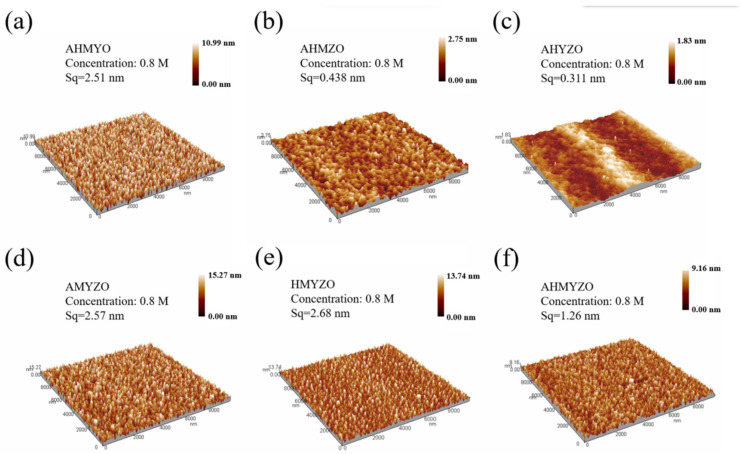
AFM images of 0.8 M groups (**a**) AHMYO; (**b**) AHMZO; (**c**) AHYZO; (**d**) AMYZO; (**e**) HMYZO; (**f**) AHMYZO.

After that, atomic force microscopy (AFM) was used to observe the surface morphologies and measure the roughness of the films. The results are shown in Figure 5 and Figure 6.

From Figure 5, we find that the roughness is larger at higher concentrations, which is probably caused by the unstable formation of atomic agglomeration when the concentration goes up [42,43]. The films have the smallest roughness at 0.6 M.

The roughness of the HMYZO film under 1.0 M is large. Meanwhile, the AHMZO and AHYZO films have better surface uniformity. During the annealing process, a metal–oxygen–metal compact structure is formed after the evaporation of the organic ligands [18]. The interaction of metal ions can lead to different metal ion arrangements during the evaporation of organic ligands, resulting in different densities of films [7,44].

**Figure 7:**
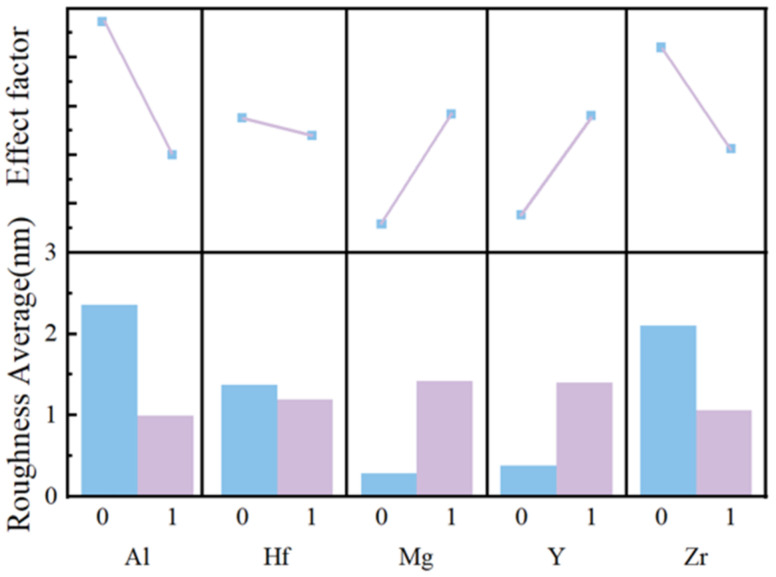
Main effect diagram of different elements on average roughness.

In order to learn the important role of various elements on surface roughness, we used Minitab to analyze the influence factor. The results are shown in Figure 7. It can be seen from Figure 7 that aluminum ions and zirconium ions can reduce surface roughness greatly. The inclusion of aluminum nitrate and zirconium nitrate can possibly reduce the contact angles of the precursors on the ITO substrate. The large contact angle of the solutions easily forms pinholes during the spin coating process. The surface roughness of films can be increased by pinholes [45].

Films with Y and Mg have larger surface roughness. Y ions have the largest ionic radius, which may lead to the increase of pores in the films. As a result, adding yttrium oxide is likely to increase surface roughness. The results are consistent with the above analysis of roughness. For Mg, this may be because Mg has a small electronegativity. The bonding between the metal atoms is relatively loose, which leads to the increase of surface roughness after adding Mg ions.

Moreover, the surface roughness of the 0.6 M AHYZO films is only 0.290 nm, and the smooth surface can reduce the formation of conductive channels and thus reduce the leakage current [46]. The good surface flatness of thin films provides a prerequisite for obtaining high electrical properties with good interlayer contact [47,48].

**Figure 8:**
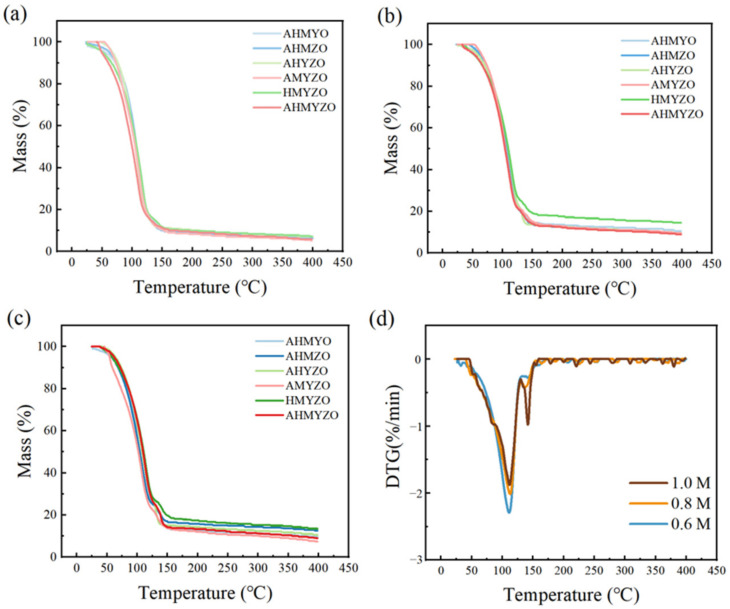
Thermogravimetric curves of thin films under (**a**) 0.6 M; (**b**) 0.8 M; (**c**) 1.0 M group; (**d**) DTG curves of AHMYO films at different concentrations.

In order to speculate on the possible chemical reactions during the annealing process, we carried out a thermogravimetric analysis of the solutions. The results are shown in Figure 8a–c. Every group has an obvious mass loss from 50 °C to 125 °C. According to the results, we infer that this is caused by the rapid evaporation of the solvent [49]. We can see that the evaporation temperature of the solution was lower than the boiling point of EGME (125 °C), which was due to the presence of various solutes in the solutions. There was another steady mass loss after the temperature reached 175 °C, owing to the movement of oxygen from the solution system in the form of CO, CO_2_, O_2_, and H_2_O [50]. In addition, the saturated vapor pressure of the liquid increases when the concentration grows, leading to the rise of termination temperature in TG curves [51]. This may be caused by the stronger binding of the solute to the solvent as the concentration increases. Therefore, the temperature required to reach equilibrium is higher.

**Figure 9:**
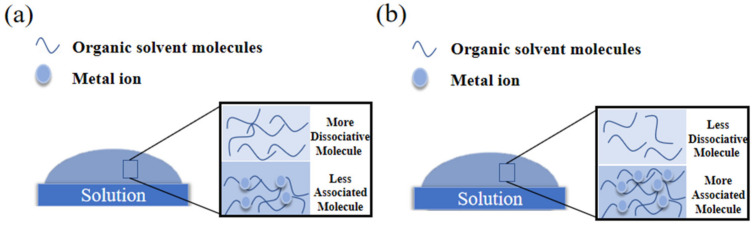
Solution molecular diagram during annealing (**a**) Precursors with low concentrations; (**b**) precursors with high concentrations.

Moreover, DTG curves of AHMYO films at different concentrations are shown in Figure 8d. As the concentration increased to 1.0 M, two peaks appeared in the DTG curves, which corresponded with the two-period decomposition. A small peak can be observed in the range of 125 °C to 175 °C under concentrations of 1.0 M [52], which means a second decomposition of the solution exists. However, similar phenomena are not obvious at 0.8 M and 0.6 M. The combination of solvent molecules and solute molecules leads to this result. It can be seen from Figure 9 that organic solvent molecules mainly exist in two forms. The number of dissociative molecules and associated molecules varies at different concentrations. Dissociative molecules are easy to evaporate, while the binding molecules are not. In addition, at high concentrations, more metal ions lead to the increase of binding molecules. The solvent can be re-evaporated only when sufficient energy is provided by increasing temperature.

From Figure 8, it can also be seen that HMYZO films have a strong binding ability to the solvent, whose final residual mass is the largest under the higher concentrations. Moreover, we find that films at high concentrations have more defects because of the greater residual mass, which shows the same results with surface roughness.

Furthermore, by comparing the TG curves of the three concentrations, there are significant differences at high concentrations. We speculate that the solution properties influence the chemical reaction in the heating process when the concentration goes up [53].

**Figure 10:**
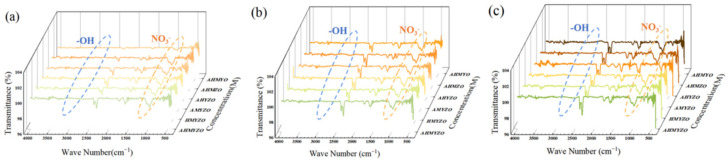
Fourier infrared transmittance curves of the films in each system (**a**) 0.6 M group; (**b**) 0.8 M group; (**c**) 1.0 M group.

We analyzed the composition of the films by Fourier transform infrared spectroscopy (FTIR). Figure 10 shows the results. IR spectral ranges of hydroxyl and nitrate are 3000–3500 cm^−1^ and 1200–1500 cm^−1^, respectively [54,55], and it can be seen from the results that nitrate and hydroxyl can be substantially removed after annealing. We also observe absorption peaks in the infrared spectrum of 2800–2900 cm^−1^, 2100–2300 cm^−1^, and 1300–1500 cm^−1^, which may be owed to a small number of acetate residues originating from acetic acid, which we added in the precursor to suppress the hydrolysis of metal oxides. The intensity of absorption peaks rises slightly with concentration going up because the residual organic content increases slightly as the concentration increases. Increased organic content will have a bad influence on leakage current. The results in Figure 10 are consistent with the analysis of organic residues in the thermogravimetric curves.

**Figure 11:**
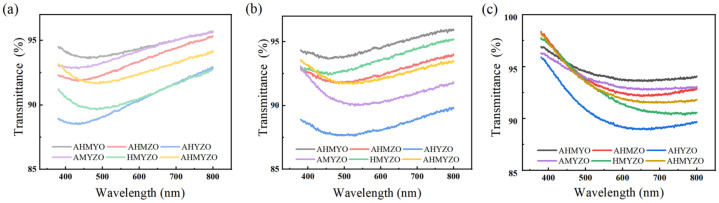
The transmittance of each concentration group films in the visible range (**a**) 0.6 M group; (**b**) 0.8 M group; (**c**) 1.0 M group.

After that, the transmittance of the films was measured by a UV-Vis spectrophotometer in the visible light range, and the results are shown in Figure 11. From the previous research, it can be seen that transparent capacitors with good performance can be made when the transmittance of the dielectric films is more than 80% [56]. In our experiment, most films can achieve a transmittance of no less than 90% in the visible light range, so these films have the potential for the preparation of transparent devices.

### 3.2. Electrical Properties

**Figure 12:**
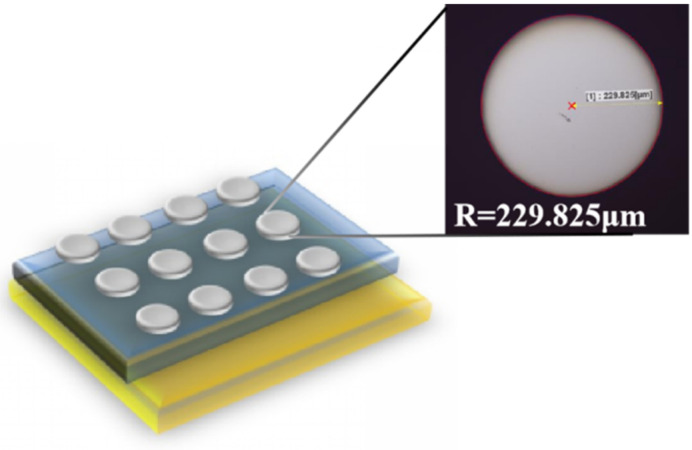
Schematic images showing MIM capacitor structure.

Metal–insulator–metal (MIM) devices were fabricated by coating thin films on ITO substrates and depositing aluminum electrodes on the dielectric layers to characterize the electrical properties and performed tests through a semiconductor analyzer. A structure of the MIM capacitor devices, illustrating the basic material stacking order, is depicted in Figure 12. A laser scanning confocal microscope was used to measure the radius of the Al electrode. It can be seen from Figure 12 that the radius of the Al electrode is 229.825 μm. Then, we calculated the capacitance density and leakage current density of each film with measured data.

**Figure 13:**
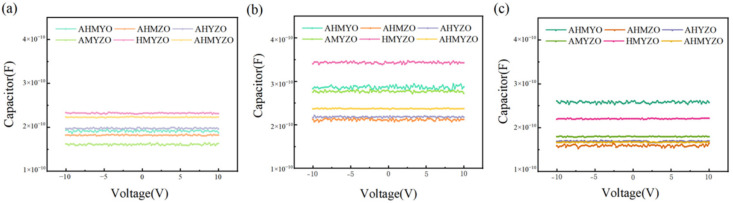
Relationship curves between capacitance and voltage (**a**) 0.6 M group; (**b**) 0.8 M group; (**c**) 1.0 M group.

Figure 13 and Table 2 show the C–V curves and capacitance densities of multi-component oxide films. It can be seen that the capacitance densities of multi-element oxide films are of high values and have good voltage stability. From Table 2, the 0.8 M group has the largest capacitance densities; we speculate that the synergistic effect of different metal ions and the film thickness jointly lead to this phenomenon. The strongest charge storage capacity of the 0.8 M HMYZO film can be seen. According to the results of thermogravimetry, we speculate that the strong chare capacity of HMYZO films comes from the most organic residual, such as hydroxyl [57]. The charge storage capacity of different films is different at the same concentration, which could be ascribed to the different interactions between molecules.

**Figure 14:**
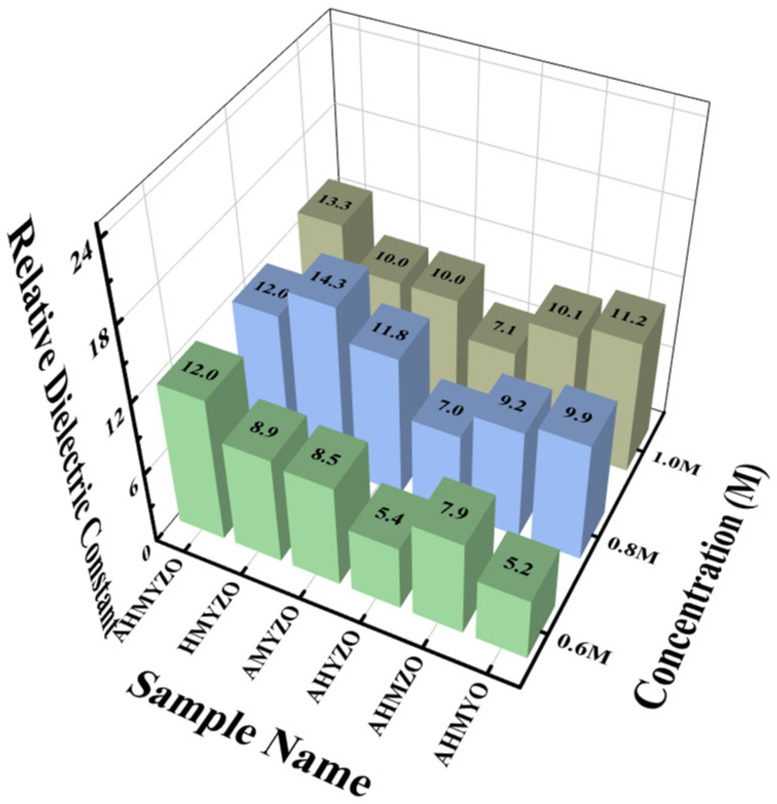
Relative dielectric constants of multi-oxide films at different concentrations and different components.

The relative dielectric constant can be calculated, and the calculation results are shown in Figure 14.

Overall, the relative dielectric constant is larger when the concentration increases. It can be seen from Figure 14 that the HMYZO of 0.8 M reaches 14.3. This system has a high relative dielectric permittivity, possibly owing to the processing of residual water and hydrogen content on AlO_x_-containing films at low temperatures beside the low relative dielectric constant of alumina [58]. Compared with the single metal film, which can only make a breakthrough in one aspect in previous studies, the dielectric films in this study not only have higher capacitance density but also can maintain low leakage current with good stability.

**Figure 15:**
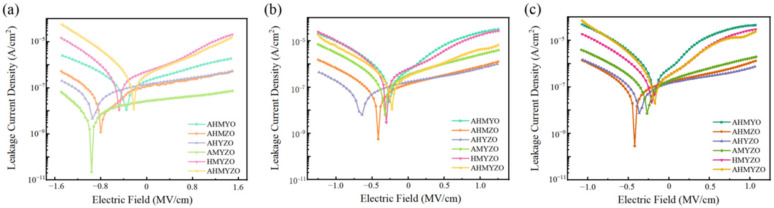
Relationship between leakage current density and field strength (**a**) 0.6 M group; (**b**) 0.8 M group; (**c**) 1.0 M group.

The I–V results are shown in Figure 15. It is found that the apparent difference in the leakage current of each combination is due to the various properties of films caused by metal interactions. The leakage currents density of the AHMYO, HMYZO, and AHMYZO films are large. Combined with Figure 5, this might be ascribed to the large roughness. When roughness increases, the leakage current rises [46,59]. The general trend shows that the leakage current increases with higher roughness. However, it can be seen that the surface roughness of AMYZO films is large while the leakage current is low. Many factors contribute to the leakage current, such as microstructure, grain size, crystallization, and defect density [44,60,61,62]. We speculate that the chloride ion hinders the combination of oxygen and hafnium, leading to more defects [63]. This phenomenon is considered to be related to different oxide film formation mechanisms [64]. Thus, the leakage current of groups with hafnium chloride goes up [65].

What is more, the leakage current increases when the concentration goes up. It is due to more defects being produced when the concentration of precursor rises as more organic residues exist. For thicker films, the increase in the number of pinholes will lead to higher body defect concentration, thus affecting the charge transfer performance [42].

The AMYZO films can achieve a small leakage current of less than 5.03 × 10^−8^ A/cm^2^ @ 1.0 MV/cm. The AHYZO and AHMZO films display stable electrical properties at different concentrations. The leakage currents of the AHYZO films range from 3.25 × 10^−7^ A/cm^2^ @ 1.0 MV/cm to 6.88 × 10^−7^ A/cm^2^ @ 1.0 MV/cm as concentrations rise. Therefore, there is more potential for multi-component metal oxide.

**Figure 16:**
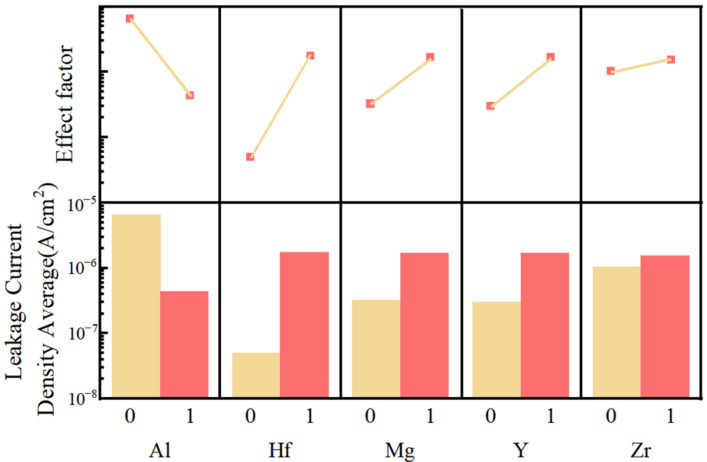
Main effect diagram of different elements on mean leakage current density.

To further analyze the effect of each element on leakage current, we used Minitab to analyze the influence factor. Figure 16 shows the results. The calculation results show that the aluminum element has the effect of significantly reducing the leakage current of the films. In addition, other elements will increase the leakage current of the films, among which the Hf has the most obvious effect. The calculation results of the impact factor correspond to the leakage current test analysis. Chloride ions from hafnium chloride are difficult to remove completely, resulting in significant growth in the leakage current of the films [57]. Therefore, the AMYZO film has the smallest leakage current. Moreover, Al ions have a good influence on leakage current. The reason is that aluminum ions can reduce the roughness of the films. In addition, for alumina, space charges are difficult to convey due to its large bandgap. The leakage current in the HMYZO films is higher than that in groups with alumina [66,67]. These analysis results are well-corresponded with the measurement of leakage current.

**Figure 17:**
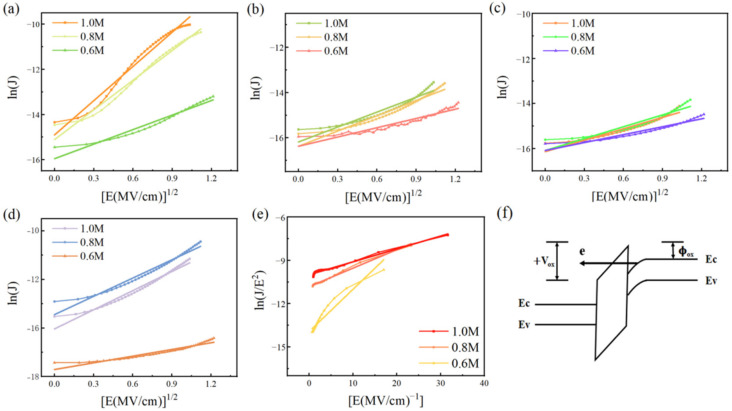
(**a**–**d**) Show the Schottky thermal emission current: the linear fitting result of ln(J) with the changing trend of E^1/2^ (**a**) AHMYO; (**b**) AHMZO; (**c**) AHYZO; (**d**) AMYZO; (**e**) Fowler–Nordheim tunneling current fitting results of AHMYO film: linear fitting results of ln(J/E^2^) changing trend with E^−1^; (**f**) Fowler–Nordheim tunneling current schematic.

To explain better the reason why the leakage current of different multi-element oxide films was different, we also fitted the leakage current density with four leakage current mechanisms. We want to confirm the dominant mechanism of each film by comparing the similarities between experimental data and the theoretical model through a linear fitting. Some results are shown in Figure 17. The conduction of leakage current can be mainly described by Schottky thermal emission (SE) [30], space charge limited current (SCLC) [13], Poole-Frenkel emission (PF) [68], and Fowler–Nordheim tunneling (FN) [69].

We find that AHMZO, AHYZO, and AMYZO films with lower leakage current correspond with the Schottky thermal emission current model. Schottky thermal emission current refers to the current formed by the injection of carriers due to the decline of the potential barrier between the electrode and the dielectric layer under the action of the electric field. The current is limited by the height of the interface barrier and the applied voltage. Therefore, we conjecture that there are fewer internal defects in AHMZO, AHYZO, and AMYZO films [70]. However, with the increase in concentration, the test curves slightly deviate from the fitting curve, as more disordered defects exist under higher concentrations. For the other three kinds of multi-element oxide films, we find that the leakage currents of HMYZO and AHMYZO films are unable to match the fitted curves of the four mechanisms, indicating that there is more than one type of defect in these two groups. AHMYO film coincides well with the FN model at high concentration. The generation principle of the Fowler–Nordheim tunneling current can be seen according to Figure 17f. Due to the large roughness and relatively small thickness of AHMYO films at higher concentrations, a stronger electric field is produced under the same voltage (V_ox_), reducing the dielectric barrier width (φ_ox_) and more tunneling current [71,72].

The AMYZO film of 0.6 M has the best insulation properties, and the leakage current density is only 5.3 × 10^−8^ A/cm^2^ @ 1.0 MV/cm. For the capacitance test, the HMYZO film obtains the maximum capacitance density, reaching 208 nF/cm^2^ at 0.8 M. The strong charge storage capacity is related to the residual polar groups, such as hydroxyl in the film. However, these groups lead to the formation of conductive channels, so its leakage current density is large. Under 1.0 MV/cm field strength, the leakage current density is 1.9 × 10^−5^ A/cm^2^. The AMYZO film has the best comprehensive performance, with low leakage current and high capacitance density (169 nF/cm^2^).

## 4. Conclusions

In this study, we prepared multicomponent oxide films with high electrical properties by solution method and low annealing temperature at 300 °C. The AHMYO film with the best optical properties has an average transmittance of 95%. In terms of leakage current density, AHYZO film has the most stable insulation performance. The leakage current density remained low at different concentrations and increased from 3.3 × 10^−7^ A/cm^2^ @ 1.0 MV/cm to 6.9 × 10^−7^ A/cm^2^ @ 1.0 MV/cm from 0.6 M to 1.0 M. According to the results, the AMYZO group at 0.6 M showed the lowest leakage current density of 5.03 × 10^−8^ A/cm^2^ @ 1.0 MV/cm and a higher capacitance density (169 nF/cm^2^). To sum up, the most prominent solution system in our study is the AMYZO group. Not only does it have ultra-low leakage current, but it also has a high capacitance density.

By combining the influence factor and leakage current mechanism analysis, we studied the role of different elements in film property and MIM device performance. Adding Y or Mg ions increases the roughness and leakage current. Al and Zr ions greatly reduce the roughness. Hf ion has little effect on roughness, but the leakage current of the Hf ion group increases obviously. For the leakage current mechanism, the larger roughness and the AHMYO film with smaller thickness cause the leakage current to be dominated by FN tunneling. The leakage currents of HMYZO films are large and unable to match the fitted curves of the four mechanisms, which is due to the combined effect of roughness and polar groups.

We find that multi-component metal oxides need to consider the compatibility between elements. Based on the measurement mentioned above, the effect of Y and Mg on the film are similar, as well as the Al and Zr. Combining the two elements with similar effects will produce better synergy. Moreover, Hf has the negative effect of increasing roughness and leakage current. Therefore, AMYZO films had the best performance.

Multi-component films prepared by simple, environment-friendly, and low-temperature solution methods are supposed to be used to prepare dielectric layers with high stability, low leakage current density, and high capacitance density. Moreover, a wide range of applications can be made, such as transparent capacitors and electronic components of small size. We believe that the preparation of multiple oxides by solution method has great research prospects.

## Figures and Tables

**Table 1:** Sample names of different precursors system.

Sample Names	Composition of Metal Oxides
AHMYO	Aluminum-Hafnium-Magnesium-Yttrium-Oxide
AHMZO	Aluminum-Hafnium-Magnesium-Zirconium-Oxide
AHYZO	Aluminum-Hafnium-Yttrium-Zirconium-Oxide
AMYZO	Aluminum-Magnesium-Yttrium-Zirconium-Oxide
HMYZO	Hafnium-Magnesium-Yttrium-Zirconium-Oxide
AHMYZO	Aluminum-Hafnium-Magnesium-Yttrium-Zirconium-Oxide

**Table 2:** Capacitive densities of 0.6 M, 0.8 M, and 1.0 M group (nF/cm^2^).

	AHMYO	AHMZO	AHYZO	AMYZO	HMYZO	AHMYZO
0.6 M	116	111	119	140	104	135
0.8 M	173	128	131	208	167	144
1.0 M	156	104	102	133	109	103

## Data Availability

Dates are contained within the article.
